# Further Insights into Brevetoxin Metabolism by *de Novo* Radiolabeling

**DOI:** 10.3390/toxins6061785

**Published:** 2014-06-10

**Authors:** Kevin Calabro, Jean-Marie Guigonis, Jean-Louis Teyssié, François Oberhänsli, Jean-Pierre Goudour, Michel Warnau, Marie-Yasmine Dechraoui Bottein, Olivier P. Thomas

**Affiliations:** 1 Institut de Chimie de Nice-PCRE (Processus Chimiques et Radiochimiques dans l’Environnement), UMR 7272 CNRS, Université de Nice Sophia-Antipolis, Faculté des Sciences, Parc Valrose Nice 06108, France; E-Mail: kevin.calabro@unice.fr (K.C.); 2 Plateforme “Bernard Rossi”—Laboratoire TIRO (Transporteur en Imagerie Radiothérapie et Oncologie), UMR E 4320 CEA /iBEB /SBTN-CAL, Université de Nice Sophia Antipolis, Faculté de Médecine, 28 Avenue de Valombrose, Nice 06107, France; E-Mail: jean-marie.guigonis@unice.fr; 3 Radioecology Laboratory, International Atomic Energy Agency—Environment Laboratories, MC 98012, Monaco; E-Mails: J.Teyssie@iaea.org (J.-L.T.); F.R.Oberhaensli@iaea.org (F.O.); M.Warnau@iaea.org (M.W.); 4 Geoazur Laboratory, Université de Nice—Sophia-Antipolis, UMR 7329 CNRS, UR 082 IRD, Campus Azur CNRS Bât. 1, 250 rue Albert Einstein, Sophia Antipolis Valbonne 06560, France; E-Mail: jean-pierre.goudour@unice.fr (J.-P.G.)

**Keywords:** *Karenia brevis*, polyketide, brevetoxins, metabolism, radiolabeling

## Abstract

The toxic dinoflagellate *Karenia brevis*, responsible for early harmful algal blooms in the Gulf of Mexico, produces many secondary metabolites, including potent neurotoxins called brevetoxins (PbTx). These compounds have been identified as toxic agents for humans, and they are also responsible for the deaths of several marine organisms. The overall biosynthesis of these highly complex metabolites has not been fully ascertained, even if there is little doubt on a polyketide origin. In addition to gaining some insights into the metabolic events involved in the biosynthesis of these compounds, feeding studies with labeled precursors helps to discriminate between the *de novo* biosynthesis of toxins and conversion of stored intermediates into final toxic products in the response to environmental stresses. In this context, the use of radiolabeled precursors is well suited as it allows working with the highest sensitive techniques and consequently with a minor amount of cultured dinoflagellates. We were then able to incorporate [U-^14^C]-acetate, the renowned precursor of the polyketide pathway, in several PbTx produced by *K. brevis*. The specific activities of PbTx-1, -2, -3, and -7, identified by High-Resolution Electrospray Ionization Mass Spectrometer (HRESIMS), were assessed by HPLC-UV and highly sensitive Radio-TLC counting. We demonstrated that working at close to natural concentrations of acetate is a requirement for biosynthetic studies, highlighting the importance of highly sensitive radiolabeling feeding experiments. Quantification of the specific activity of the four, targeted toxins led us to propose that PbTx-1 and PbTx-2 aldehydes originate from oxidation of the primary alcohols of PbTx-7 and PbTx-3, respectively. This approach will open the way for a better comprehension of the metabolic pathways leading to PbTx but also to a better understanding of their regulation by environmental factors.

## 1. Introduction

**Figure 1:**
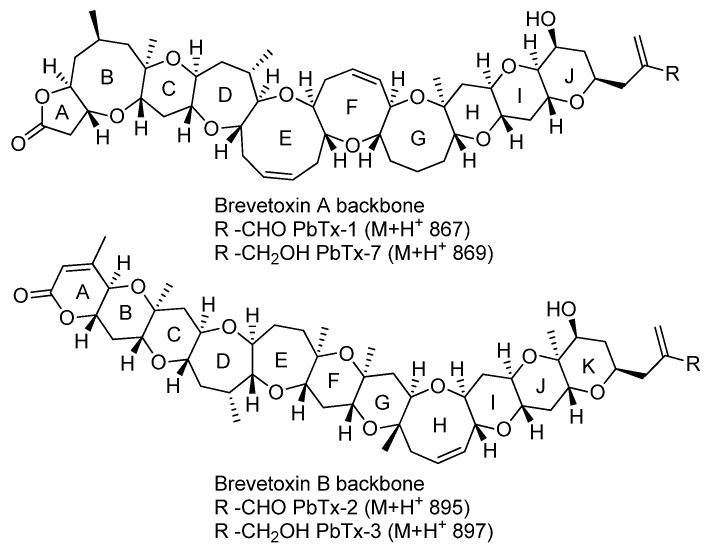
Two chemical families of brevetoxins.

Brevetoxins (PbTxs) are potent neurotoxins produced by *Karenia brevis* (*K. brevis*), a dinoflagellate responsible for recurrent harmful algal blooms in the Gulf of Mexico. PbTxs have been found to affect marine ecosystems, being associated to massive fish kills, bird deaths or marine mammal mortalities, and may induce human food or respiratory intoxication through the bioaccumulation of the toxins by comestible shellfishes or aerosols [1,2]. The chemical diversity produced by this dinoflagellate is far from being exhaustively described and new metabolites are regularly reported from this species [3,4]. Toxic brevetoxins are chemically separated into two families featuring PbTx-A and PbTx-B backbones, with PbTx-1 and PbTx-2 as key representatives of both families, respectively (Figure 1) [5]. Currently, 12 polyether derivatives have been isolated from *K. brevis* [6,7,8,9,10,11,12,13,14].

Environmental changes have been shown to dramatically modify the metabolic profiles of this micro-organism [15]. The composition in brevetoxin congeners has been shown to vary over the growth of *K. brevis*, in culture and in natural blooms [16], with PbTx-2 as the major toxin during the log phase and PbTx-3 and PbTx-1 increasing during the stationary phase. It is also known that the chemical composition of the algae depends on the strain used and, consequently, a large part of the chemodiversity still remains to be discovered [17,18,19]. In order to better understand and possibly control the production of toxins in the environment, their metabolic pathways should be unraveled [20,21]. Even if the whole pathway of these complex polyethers has not been fully elucidated, a polyketide origin has been demonstrated and the biosynthetic genes have been under acute investigation during the last decade [16,22,23,24]. In this context, feeding experiments with labeled precursors are the first steps giving key information on the construction of complex natural products. Such experimental data were obtained in the 1980s, leading to some controversy [11,25,26,27]. The main conclusions underlined a high originality in the biosynthesis of these marine toxins through a larger involvement of citric acid cycle and the incorporation of succinate and α-ketoglutarate instead of propionate units. Stable isotope feeding studies found unusual truncations of acetyl groups in brevetoxin, as well as methyl side chains, derived from methionine, that indicate brevetoxin biosynthesis may require atypical polyketide synthases [11,25,26]. Even with the last results obtained from molecular biology, their whole metabolic pathway has not been totally unraveled to date, and this issue clearly deserves additional experimental data.

Due to the development of highly sensitive and resolutive analytical tools, the detection of low incorporation rates (measure of isotopic ratio) has become possible and we decided to undertake some feeding experiments with this dinoflagellate using the most sensitive techniques for detection [28]. In addition to giving valuable information on the biochemical transformations occurring in living organisms, such *in vivo* experiments mostly traduce the *de novo* metabolic activities of the cells and hence the direct response of the organism to environmental changes. We consequently decided to develop a very sensitive and general process for the feeding experiments of dinoflagellates with radiolabeled precursors, enabling the measure of the specific activity of several metabolites by a straightforward HPLC purification/RadioTLC detection [29]. This highly sensitive process allows us to work with close to “natural” concentration of precursors.

## 2. Results and Discussion

PbTx-2 is the most abundant metabolite usually produced by *K. brevis* in culture, and is commercially available. We, hence, decided to set up our feeding protocol with [U-^14^C]-acetate as a radiolabeled precursor and this toxin as our main target. We performed five feeding experiments. A first set of experiments (A0, B0 and C0) inoculated together with increasing concentrations of [U-^14^C]-acetate without antibacterial agent. The second set of experiments was performed with addition of antibacterial agents just after the exponential stage followed by feeding of two different concentrations of the same acetate precursor the next day (A and B). These experiments allowed us to assess the effect of the concentration of acetate on the metabolomic profile. The decision to add some antibacterial agents in the medium was made because cell death was observed with higher concentration of acetate. Additionally, quantification of the specific activity of the labeled compounds in experiment B afforded some information on the controversial metabolic pathway of these toxins.

### 2.1. Culture Growth Rates

The growth rates of *K. brevis* NOAA1 strain were measured in each experimental condition.

In the first set of experiments performed in 1 L f_10k_ medium, the control culture exhibited a growth rate of 0.29 div·day^−1^. With an initial concentration of [U-^14^C]-acetate of 100 kBq·L^−1^ (0.026 µm, A0), the rate was 0.31 div·day^−1^. This rate decreased to 0.07 div·day^−1^ when the concentration of acetate was raised to 500 kBq·L^−1^ (0.13 µm, B0) and a rapid death of the culture (within three days after inoculation) was observed with a concentration of 1000 kBq·L^−1^ (0.26 µm, C0). As previously reported, the increase in acetate concentration induced a bacterial proliferation and consequently a decrease in the pH from 8.1 to 7.6, which is unsuitable for the algal culture [11]. Because we anticipated that working at the A0 concentration would lead to a very low specific activity of the targeted products, we decided to perform the second set of experiments in the presence of antibacterial agents allowing working with higher concentrations of acetate (A × 2 and B × 5).

In the second set of experiments performed in 2 L volume media where antibiotics penicillin G and streptomycin were added at day 26 (at the end of the exponential stage) and the cultures fed with [U-^14^C]-acetate, 200 kBq·L^−1^, (0.052 µm, A) and [U-^14^C]-acetate, 500 kBq·L^−1^ (0.13 µm, B) respectively, the growth rates were 0.17 ± 0.02; 0.16 ± 0.01 div·day^−1^. In this case, neither significant change in the pH nor cell death was observed in the culture media, confirming our hypothesis on the bacterial proliferation inducing cell death. The repeatability of the growth curve was rather satisfactory for each experiment, the lag phase ended 12 days after inoculation and the subsequent exponential phase lasted 15 days.

The difference of growth rates between the two sets of experiments is explained by the volume culture. For the first set of experiments including the control, cultures were grown in 1 L culture media while, for the second set of experiments, cultures were grown in 2 L culture media. Both sets were inoculated with the same cell concentration.

We further decided to control changes in the metabolic profiles during these experiments.

### 2.2. Metabolomic Profiling

**Figure 2:**
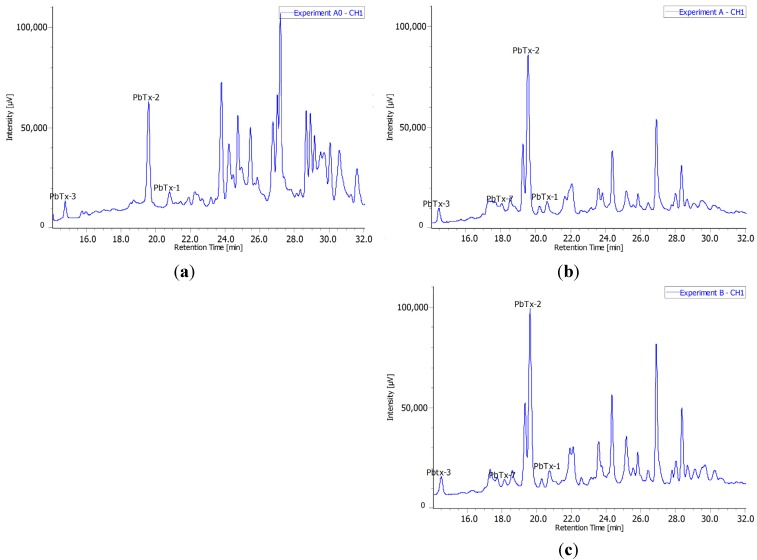
Metabolomic profiles of *K. brevis* crude extracts: (**a**) [U-^14^C]-acetate 100 kBq·L^−1^ spiked day 0 (Experiment A0); (**b**) [U-^14^C]-acetate 200 kBq·L^−1^ spiked after exponential stage (Experiment A); (**c**) [U-^14^C]-acetate 500 kBq·L^−1^ spiked after exponential stage (Experiment B), on a phenyl-hexyl column at 215 nm.

Clear differences were observed in the metabolomic profiles of both sets of experiments. Experiment A0 was performed in close to “natural conditions” with low concentration of acetate (0.026 µm) without antibiotics. In the second set of experiments A and B with antibiotics and higher concentrations of acetate, we were far from “natural” conditions and the metabolism was clearly affected (Figure 2). Because the acetate concentrations remain below 0.13 µm, we hypothesize that the effect on the profiles may rather be inferred to the presence of the antibiotics added into the culture medium after the log phase. Interestingly, both profiles of the second set of experiments were very similar, thus, confirming the change in the metabolism in these conditions.

**Figure 3:**
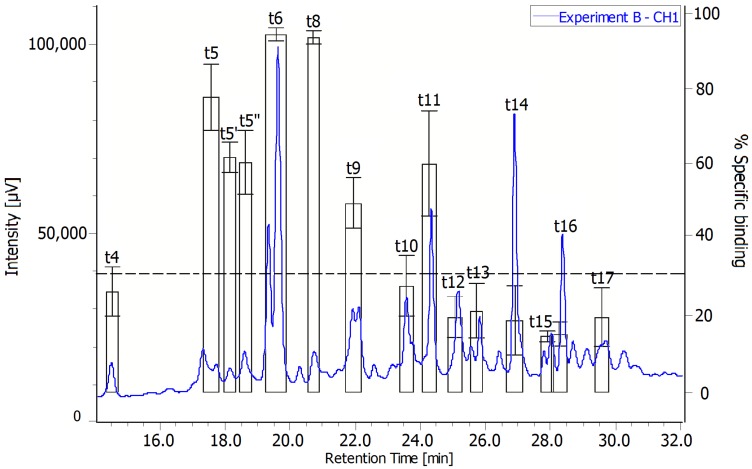
RBA on fractions obtained from extract B.

The next step was to identify the structures of the toxins present in the extract. The extract obtained from experiment B performed with the highest concentration of acetate was first fractionated by reverse-phase HPLC on a PhenylHexyl column. To locate the brevetoxins in the metabolomic profile, we first applied the Receptor Binding Assay to the obtained fractions (Figure 3). We considered the presence of brevetoxins in a fraction when the percentage of specific binding exhibited a value higher than 30. Among the 33 fractions collected by HPLC, eight fractions absorbing significantly at 215 nm in UV, exhibited a percentage of specific binding higher than the limit value. The toxic fractions found in the first 25 min of the metabolomic profiles were further analyzed by High-Resolution Electrospray Ionization Mass Spectrometry (HRESIMS) for structure identification.

PbTx-2 was easily located in fraction t6 by coelution with a commercial standard and additional HRESIMS analysis (*m*/*z* 895.4841 [M + H]^+^ Δ + 0.33 ppm). A second purification step on a C18 column was necessary to isolate pure PbTx-2 from the t6 fraction, the first coeluting peak being inactive in the RBA. PbTx-3 was identified in t4 without any further purification (*m*/*z* 897.4999 [M + H]^+^ Δ + 0.49 ppm). On the basis of HRESIMS data, we were also able to identify PbTx-1 (*m/z* 867.4941 [M + H]^+^ Δ + 5.9 ppm) and PbTx-7 (*m*/*z* 869.5050 [M + H]^+^ Δ + 0.54 ppm) in t8 and t5’ respectively.

All four toxins were quantified by HPLC-UV in the different experiments. For PbTx-2, 12.3, 14.2, and 11.9 were produced by A0, A, and B cultures respectively. In consequence, one cell of this strain produced 1.00 ± 0.08 pg of PbTx-2 which is a common value for *K. brevis* culture [5]. The presence of antibacterial compound or changes in the concentration of acetate did not induce any significant effect on the toxin production by the alga. The same observation was made for PbTx-3 and PbTx-1 while PbTx-7 was not detected in the A0 culture. Additional observations have been made for the A0 experiment: the non-toxic coeluting peak in t6 was absent and several low toxic compounds eluting above *t*_R_ 25 min were much more expressed.

In order to gain insights into the metabolic pathways, the specific activities of the four identified toxins were assessed for experiment B corresponding to the highest concentration of acetate.

### 2.3. Radiochromatograms and Specific Activity

**Figure 4:**
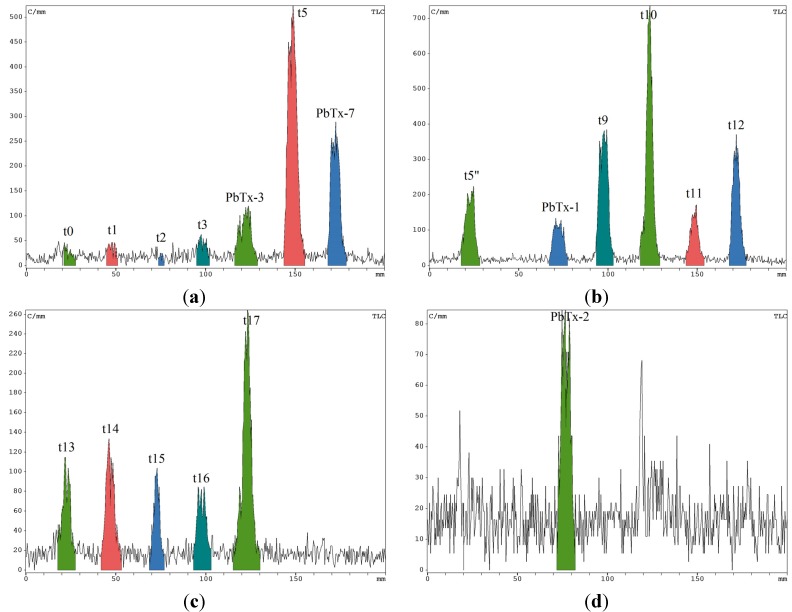
RadioTLC counting of (**a–c**) PhenylHexyl HPLC fractions from culture B; (**d**) t6 fraction (PbTx-2) after purification on C18.

The specific activities of the four compounds were assessed by HPLC-UV and RadioTLC counting. The fractions collected by HPLC from culture B extract were evaporated and deposited on a TLC plate. The radioactivity associated to each fraction was counted by the very sensitive RadioTLC (Figure 4a–c) [28]. Because t6 was a mixture, we first performed a C18 HPLC purification before depositing purified PbTx-2 (Figure 4d). The radioactivity of the purified compound was very similar to the radioactivity present before the second purification step and cross-purification on different static phase in HPLC precluded any possible contamination of our process. This can mainly be explained by the low level of radioactivity used in each experiment (below 1000 kBq).

The radiochromatogram clearly evidenced that [U-^14^C]-acetate has been incorporated in several compounds present in the metabolomic profile. In addition to PbTxs (t4, t5’, t6, and t8), other compounds highly incorporated radiolabeled acetate. HRESIMS did not allow the identification of these highly radiolabeled compounds because the detected mass did not correspond to already known metabolites from this species but several explanations can be found for this high incorporation. First, highly radiolabeled compounds, such as t9, t10, and t12, may be intermediates in the biosynthesis of PbTxs. Alternatively, these rather non polar non identified metabolites may originate from other metabolic pathways involving acetate as a precursor like usual fatty acid pathway. Indeed, recent studies evidenced the presence of fatty acid synthases in *K. brevis* [22].

We were then able to assess the specific activity of the four identified compounds in culture B (Table 1). In order to compare with close to natural conditions, we also assessed the specific activity of PbTx-2 from A0 culture.

The results with PbTx-2 are intriguing as the quantity of toxin produced in both experiments A0 and B are very similar while the specific activity in experiment B is around five times lower than for experiment A0. This is still more intriguing considering that the initial concentration of the radiolabeled precursor was five times higher in experiment B. Consequently, a higher specific activity was expected in this case. Because the quantity of PbTx-2 was rather similar in both experiments, we cannot infer this discrepancy to the concentration of acetate, nor the presence of antibacterial compounds. The only rational explanation would stand in the moment of acetate spiking. Indeed, in the case of experiment A0, acetate was spiked at the beginning of the experiment, while acetate was added to culture B at the end of the log phase. This result highlights some key features on feeding studies with labeled precursors. First, the use of radiolabeling rather than giving a static view of the quantity of toxins present in the cells, enables the observation of the *de novo* activities of the dinoflagellate, working in close to “natural” conditions. Indeed, high isotopic ratio and then specific activities are related with a very dynamic process. These kinetic data are essential in order to fully understand and control the metabolic pathways of these important toxins. The higher specific activity observed in A0 culture suggested that the *de novo* production of toxins from acetate started before the end of the log phase.

**Table 1:** Specific activities of the four identified brevetoxins.

Experiment	Toxin	Quantity (nmol)	Radioactivity (Bq)	Specific Activty (Bq·nmol^−1^)
A0	PbTx-2	18	55	3.1
B	PbTx-2	13	8.9	0.67
	PbTx-3	9.7	15	1.5
	PbTx-1	1.9	15	7.9
	PbTx-7	2.5	27	11

The relative values obtained for the specific activities of the alcohols PbTx-3 and PbTx-7 and the corresponding aldehydes PbTx-2 and PbTx-1 gave some important insights into the chronology events of the oxydo/reductive process involving these compounds. Indeed, in a step-by-step metabolic pathway, the specific activity of the intermediates is always expected to decrease even if a release in seawater is expected to occur. Our data would be in accordance with the fact that, in both cases, the alcohols would be intermediates in the biosynthesis of PbTx-1 and PbTx-2 and not the contrary. With specific activities being twice higher for the alcohols than for the corresponding aldehydes of both types of brevetoxins, we would be in the presence of an oxidative process converting primary alcohols into aldehydes and not a reductive process as previously stated. It is impossible to discard at this stage that these aldehydes could afterwards lead back to the alcohols by additional reductive processes as already reported. Our results are also in contradiction with the hypothesis of Shimizu stating that the aldehydes could be produced after a decarboxylative process of an epoxide [20,21]. Because our conclusions are in accordance with an oxidative process from an alcohol into an aldehyde, we suggest that the first precursor of the polyketide chain leading to brevetoxins could be a derivative of 3, 3-dimethylacrylic acid (a common C5 unit). This terminal alkyl chain could undergo an oxidative process at the allylic position of the double bond after isomerisation first leading to an alcohol and then an aldehyde, as commonly encountered in terpenoid biosynthesis.

## 3. Experimental Section

### 3.1. Cell Culture Maintenance and Counting

The strain of *Karenia brevis* (NOAA-1) used in this study was isolated by Dr. Steve Morton (NOAA, Charleston, CA, USA) from a sample collected in Port Charlotte, FL, USA. The *K. brevis* cultures were grown in an atypical f_10k_ medium [20] with salinity 33 g·L^−1^ at 23 ± 1 °C under bilateral luminosity 66–80 µE·m^−2^·s^−1^ provided on 14:10-h light/dark cycle during 3 months corresponding to 5 generations. For every generation, old *K. brevis* cultures were spiked in a new f_10k_ medium to have an initial cell concentration of 10^6^ cells·L^−1^. The seawater used in media preparation was aged Mediterranean seawater pumped from the front of Monaco bay at 30-meter’s depth. Cell counting was performed every two days in Lugol 10% using Sedgewick Rafter (20 × 50 µL) counting chamber under microscope (Nikon E200, Nikon Instruments, Tokyo, Japan).

### 3.2. Feeding with of Labeled Sodium Acetate

#### 3.2.1. First Set of Experiments

*Karenia brevis* were grown in the same conditions as described in Section 3.1 in 1 L of volume culture. Microalgae were fed the same day of their inoculation with [U-^14^C]-CH_3_COONa to have respectively in media A0, B0 and C0, a total radioactivity of 100 (0.026 µm), 500 (0.13 µm) and 1000 (0.26 µm) kBq·L^−1^ (Commercial solution of [U-^14^C]-CH_3_COONa, 1.85 MBq per vial, in ethanol, *C* = 37.0 MBq·mL^−1^, Specific Activity = 3.92 GBq·mmol^−1^, Hartmann Analytics). Cultures were harvested 18 days after inoculation. Growth rates were measured for the exponential stage in *div*·*day*^−1^ between days 5 and 15. Between these two dates the formula for growth rate is:

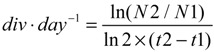
(1)
where in this case, *N*1 and *N*2 are the number of cells at day 5 and day 15 after inoculation.

#### 3.2.2. Second Set of Experiments

Cultures were grown in 2 L volumes following the same conditions as described in Section 3.1*.* 26 days after inoculation, penicillin-G (53.3 mg) and streptomycin sulfate (55.5 mg) were added in media A and B. The following day, the culture was enriched with [U-^14^C]-CH_3_COONa to have respectively, for media A and B, a total radioactivity of 200 (0.052 µm) and 500 (0.13 µm) kBq·L^−1^. After 72 h, the culture was harvested. Growth rates were measured for the exponential stage in *div*·*day*^−1^ between days 12 and 27.

We used the same formula as above where in this case, *N*1 and *N*2 are the number of cells at day 12 and day 27 after inoculation.

### 3.3. Extraction of Metabolites

The whole culture was filtered using gravity or mild vacuum pressure (<5 kPa) on 1 µm polycarbonate filters (Whatman, Little Chalfont, UK). Filters were pooled and extracted with 20 mL methanol into a 50 mL conical Falcon tube (BD Falcon™, Franklin Lakes, NJ, USA) and then stored overnight at –20 °C [5]. Filters were sonicated for 10 min (Branson, Emerson Industrial Automation, Danbury, CT, USA) and centrifuged for 4 min at 4400 g (Rotor F28/50, Sorvall, Thermo Fisher Scientific, Waltham, MA USA). All supernatants were pooled. The sonication and filtration procedure was repeated once. All supernatants were dried under nitrogen (variant pressure and temperature bath set at 40 °C) using a Turbovap LV Evaporator (Biotage, Uppsala, Sweden). The dried residue was then resuspended in 2 mL of aqueous methanol (10%). The extracts were further fractionated using C18 SPE cartridge. Briefly, the SPE cartridge (Phenomenex, Torrance, CA, USA, 500 mg, 6 mL) was conditioned with one column volume of methanol and then water, the aqueous methanolic extract was loaded onto the SPE, and washed with 2.5 volumes of distilled water. The metabolites were eluted with 5.5 mL of MeOH (100%) and the eluent was evaporated under nitrogen gas using a Turbovap LV evaporator. The dried residue was finally resuspended into 150 µL MeOH and stored at –20 °C until analysis by liquid chromatography and biochemical tests.

### 3.4. Receptor Binding Assay

Brevetoxin levels in cell extracts were quantified using the receptor binding assay following the procedure developed by Dechraoui *et al*. [30], with some modifications. Briefly, various concentration of samples were incubated for 1 h at 4 °C in the presence of ^3^H-PbTx-3 (1.5 nM) and rat brain membrane preparation in a phosphate buffered saline PBS supplemented with Tween 20 and BSA solution. The level of toxin was estimated against a standard curve of PbTx-3 obtained following similar binding conditions. The radioligand receptor binding assay was also used to guide each fraction by HPLC. For this experiment, 5 µL of each fraction were tested via the same protocol described above.

### 3.5. Analytical Methods

#### 3.5.1. Liquid Chromatography

For the chemical analysis of the *Karenia brevis* extracts, an analytical method was developed by HPLC-UV using two commercial brevetoxins produced by *K. brevis*, PbTx-2 and PbTx-3. The HPLC system used was LC-NetII/ADC Jasco (Jasco, Easton, USA). HPLC purification was performed on a semi-preparative column Luna 250 mm × 10 mm, 5 µm, Phenyl-Hexyl (Phenomenex, Torrance, CA, USA) using a mobile phase of water (A) and acetonitrile (B). The method was developed on 45 min acquisition time: 5 min 50% B followed by a linear gradient to 100% B at 25 min maintained at 100% up to 35 min. The injection volume was set at 50 µL. The flow rate of the mobile phase was 3.0 mL·min^−1^. Fractions were collected by an automatic collector (CHF122SC, Advantec, Tokyo, Japan) then concentrated and lyophilized respectively by Speedvac SPD111V (Thermo Fisher Scientific) and Lyophilizator Alpha 1-2 LD (Fisher Bioblock Scientific, Waltham, MA, USA). Finally, each fraction were resuspended in 100 µL methanol and stored into a freezer at –20 °C for Radio-TLC analysis. The fraction containing PbTx-2 was purified on a semi-preparative column SymmetryPrep™ 7.8 mm × 300 mm, 7 µm, C18 (Waters Corporation, Milford, CT, USA). The mobile phase was constituted of water (A) and acetonitrile (B). The gradient used was as follows: 2 min 50% B followed by a linear gradient to 80% B at 22 min then 95% B at 23 min maintained at 95% up to 29 min. The flow rate was 3.0 mL·min^−1^. The wavelength of the DAD was set at 215 nm.

#### 3.5.2. Identification of the Metabolite by HRESIMS

The MSI system of the LTQ-Orbitrap hybrid mass spectrometer (Thermo Fisher Scientific) was operated in the positive mode at a voltage of 5 kV, with no sheath or auxiliary gas and in maintaining the ion transfer tube at 275 °C. The capillary LC separation were performed on a Synergi 4 u Hydro-RP 80A column (250 mm × 0.30 mm, 4 µm, Phenomenex) using a mobile phase of water (A) and acetonitrile (B) with 0.1% formic acid as an additive. For identification of brevetoxins congeners the LC was operated under a gradient elution: 2 min at 50% B, linear gradient to 80% at 30 min, 95% B at 35 min for 9 min returned to 50% B at 45 min and held for 15 min before next injection. The mobile-phase flow rate was 4.2 µL·min^−1^ and the column temperature was 25 °C. The Orbitrap mass analyzer was calibrated according to the manufacturer’s directions using a mixture of caffeine, MRFA peptide, and Ultramark for positive ionization mode. MS data were acquired in full scan between 100 and 1100 uma with resolving power setting of 30 K at *m*/*z* 400. To achieve the highest possible mass accuracy, the lock mass function was enabled with the pollutant ion at an *m*/*z* of 391.28429 in the positive ionization mode used for real-time internal recalibration. MS data acquisition and processing were performed using Xcalibur software (Version 2.0.7; Thermo Fisher Scientific). Spectral accuracy was calculated by MassWorks using sClips software (Version 2.0; Cerno Bioscience, Norwalk, CT, USA). The mass tolerance for the sClips searches was 10 ppm.

#### 3.5.3. Quantification of PbTxs by UHPLC-UV

An analytical method was developed for the quantification of the toxins by UHPLC-UV with two commercial brevetoxins produced by *K. brevis*, PbTx-2 and PbTx-3. UHPLC analyses were performed on a Luna column 100 mm × 2.1 mm, 1.7 µm, Phenyl-Hexyl (Phenomenex) using a Dionex Ultimate 3000 UHPLC (Thermo Fisher Scientific) system coupled to a diode array detector. The mobile phase consisted of water (A) and acetonitrile (B). The gradient used was as follows: 2 min 50% B followed by a linear gradient to 100% B at 10 min maintained at 100% up to 12 min, then back to its original proportion 50% B until 14 min. The injection volume was set at 10 µL. The flow rate of the mobile phase was 0.43 mL·min^−1^ and the wavelength of the diode array detector was set at 215 nm. Calibration curves were obtained for both compounds PbTx-2 and PbTx-3 and the same equations were used for the quantification of PbTx-1 and PbTx-7, respectively, as they share similar chromophores.

#### 3.5.4. Quantification of the Radioactivity by Radio-Thin Layer Chromatography and Specific Activity

Incorporation of [U-^14^C]-acetate into the toxins was monitored by Radio-Thin Layer Chromatography (RITA Star β-Scanner, Raytest, Straubenhardt, German). In a typical experiment, the fractions were deposited on a 20 cm × 20 cm silica plate and radio-analyzed during one hour. For each fraction, 20 µL of the methanolic solution described above were spotted on the plate for the radio-counting. The analysis was followed in real time by Gina Star software and stooped after 1 h of counting. To assess the radioactivity in Bq of the fractions collected after HPLC, a standard curve of [U-^14^C]-proline was used. The specific activity (SA) was then assessed dividing the radioactivity by the corresponding quantity obtained by HPLC-UV.

## 4. Conclusions

Several results should be highlighted from our study. First, radiotracing with ^14^C-acetate appears as a powerful method to address the metabolic activity but also some metabolic pathways of toxic dinoflagellates producing polyether derivatives. It allows working at close to natural conditions avoiding changes in the metabolism of these microalgae induced by the presence of large quantity of precursors or antibacterial compounds. We were able to demonstrate that the metabolic activity of brevetoxins started early during the cell growth of *Karenia brevis* and this result should be taken into account for further feeding experiments. The measurement of the specific activity of the major PbTxs gave important results on the chronology of a key metabolic reaction. The specific activities of both major toxins, PbTx-1 and -2, featuring a key terminal aldehyde and representing both types of brevetoxins, were found to be twice lower than the corresponding alcohols PxTx-7 and PbTx-3. This result implies that PbTx-1 and PbTx-2 should be produced by oxidation of the allylic alcohol of PbTx-7 and PbTx-3 respectively, a result in contradiction with the accepted hypothesis that several brevetoxin congeners are derived from the aldehydes analogues. This result underlines the importance of feeding experiments with radioisotopes to assess the metabolic pathways of these complex toxins but also the chronology of the involved biochemical steps.
